# Evolutionary Diagnosis of non-synonymous variants involved in differential drug response

**DOI:** 10.1186/1755-8794-8-S1-S6

**Published:** 2015-01-15

**Authors:** Nevin Z Gerek, Li Liu, Kristyn Gerold, Pegah Biparva, Eric D Thomas, Sudhir Kumar

**Affiliations:** 1 Institute for Genomics and Evolutionary Medicine, Temple University, Philadelphia, PA 19122, USA; 2 Department of Biology, Temple University, Philadelphia, PA 19122, USA; 3 Center for Excellence in Genome Medicine and Research, King Abdulaziz University, Jeddah, Saudi Arabia; 4 Center for Evolutionary Medicine and Informatics, Biodesign Institute, Arizona State University, Tempe, AZ 85287-5301, USA

## Abstract

**Background:**

Many pharmaceutical drugs are known to be ineffective or have negative side effects in a substantial proportion of patients. Genomic advances are revealing that some non-synonymous single nucleotide variants (nsSNVs) may cause differences in drug efficacy and side effects. Therefore, it is desirable to evaluate nsSNVs of interest in their ability to modulate the drug response.

**Results:**

We found that the available data on the link between drug response and nsSNV is rather modest. There were only 31 distinct drug response-altering (DR-altering) and 43 distinct drug response-neutral (DR-neutral) nsSNVs in the whole Pharmacogenomics Knowledge Base (PharmGKB). However, even with this modest dataset, it was clear that existing bioinformatics tools have difficulties in correctly predicting the known DR-altering and DR-neutral nsSNVs. They exhibited an overall accuracy of less than 50%, which was not better than random diagnosis. We found that the underlying problem is the markedly different evolutionary properties between positions harboring nsSNVs linked to drug responses and those observed for inherited diseases. To solve this problem, we developed a new diagnosis method, Drug-EvoD, which was trained on the evolutionary properties of nsSNVs associated with drug responses in a sparse learning framework. Drug-EvoD achieves a TPR of 84% and a TNR of 53%, with a balanced accuracy of 69%, which improves upon other methods significantly.

**Conclusions:**

The new tool will enable researchers to computationally identify nsSNVs that may affect drug responses. However, much larger training and testing datasets are needed to develop more reliable and accurate tools.

## Background

Pharmaceutical drugs have been critical to maintaining global health in the 21^st^ century [1,2]. While they are frequently prescribed for patients worldwide, it is now clear that most of them are effective in only a modest fraction of the patients [3,4]. Furthermore, they may even cause adverse reactions in many people, leading to 100,000 deaths per year [5-7]. Differences in individual drug responses are due to many factors, including environment, dosage, physiological traits, and genetics [8]. Of these, the focus on genetic variants underlying differential drug response and toxicities is growing [9-11]. It is thought that a patient genetics-centric prescription may be useful to avoid ineffective treatments and side effects [12], especially because advances in DNA sequencing technology now allow for high throughput analysis of personal genomes [13-15]. In particular, exome sequencing has now become affordable and it will be useful as a first step in identifying any personal amino acid altering variants in proteins-of-interest that may influence drug response [12]. However, personal exomes are full of novel, rare variants [16], which necessitate an initial computational screening to identify candidate nsSNVs.

Computational prediction of the functional impact of nsSNVs has been routinely used in discovering variants associated with Mendelian diseases and complex diseases [17-21]. Several bioinformatic tools reported prediction accuracy as high as 89% [22-24]. Although it is intuitive to directly borrow these methods for the purpose of screening nsSNVs on their drug-response phenotypes, the performance of these tools in this specific domain is never evaluated. In fact, because these bioinformatic methods heavily rely on the evolutionary properties of nsSNVs, they will perform well only if disease-associated variants and drug-response-associated variants share similar evolutionary patterns.

Therefore, the initial focus of this study was to evaluate existing bioinformatics tools in the realm of differential drug responses. Our results indicated that there is a need for developing a new prediction model to improve the accuracy of diagnosis. We then examined the evolutionary properties (e.g., conservation profiles and the nature of mutational changes) that distinguish drug-response altering (DR-altering) from drug-response neutral (DR-neutral) nsSNVs. Based on these findings, we present our new statistical model, called Drug-Evolutionary Diagnosis (Drug-EvoD), for testing nsSNVs on their effect on drug responses. However, at the end, we pointed out that much larger training and testing datasets are needed to develop more reliable and accurate tools.

## Results and discussion

### Known drug-related nsSNVs

**Figure 1:**
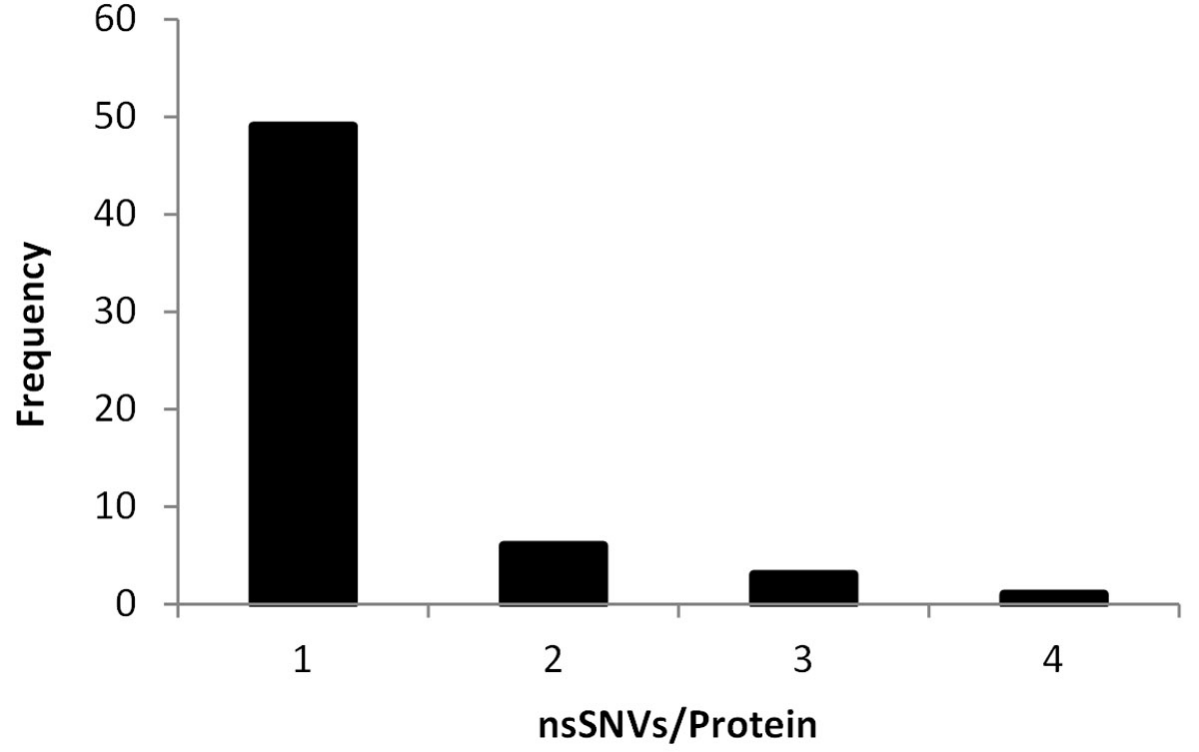
**Frequency distributions of DrugVar nsSNVs in proteins.** Majority of the proteins contain only one nsSNV that is associated with drug responses.

Pharmacogenomics Knowledge Base (PharmGKB, [2,15]) is a publicly available database dedicated to understanding how genetic variations in the human genome lead to variations in clinical responses to various drugs. It also provides integrated knowledge on relationships among genes, drugs, and diseases from clinical trials, case studies, genome-wide association studies, and functional *in vivo* and *in vitro* studies. Although over a thousand of entries are recorded in PharmGKB, most of them correspond to multiple observations involving the same nsSNVs and the same drug. Ultimately, only 263 unique nsSNVs in 178 proteins were found (see Figure S1 in Additional File 1 and Additional File 2 for the distribution of the nsSNVs across different family of proteins and corresponding data). After careful curations (see **Methods**), we identified a total of 74 nsSNVs in 59 proteins (Figure 1), for which multiple evidences supported their unambiguous effect on drug responses. This dataset (DrugVar) consists of 31 DR-affecting nsSNVs (true positives, see Table S1 in Additional File 3) and 43 DR-neutral nsSNVs (true negatives, see Table S2 in Additional File 4). It was used as the control data to test the performance of various computational tools, and served as the training data to build new statistical models. To our best knowledge, DrugVar is the first well-curated dataset depicting the genotype-phenotype relationship for drug responses.

### Effectiveness of existing methods

We chose to test the performance of two widely used methods, SIFT [24] and PolyPhen-2 [22], and a new tool that improves upon their accuracy in some cases (EvoD [23]). These prediction tools were originally designed to forecast the functional impact of an nsSNV in disease domains. However, when applied to the drug-response domain using DrugVar dataset, all three methods showed rather low overall accuracies (Table 1). Only 39% - 53% DR-affecting nsSNVs and 46% - 60% DR-neutral nsSNVs were correctly diagnosed. Their average accuracies are in the range of 44% and 52%, which is not much different from random diagnosis. This raised the doubt on the usefulness of existing tools for diagnosing nsSNVs with differential drug responses.

**Table 1:** Performance of EvoD, PolyPhen-2 and SIFT when analyzing PharmGKB variants.

	Diagnosis Rate				
	
Method	TNR	FPR	FNR	TPR	Balanced Accuracy
**EvoD**	49%	51%	61%	39%	44%
**PolyPhen-2**	60%	40%	55%	45%	52%
**SIFT**	46%	54%	47%	53%	50%

Note – TPR: True Positive Rate (sensitivity), FPR: False Positive Rate, TNR: True Negative Rate (Specificity), FNR: False Negative Rate

**Figure 2:**
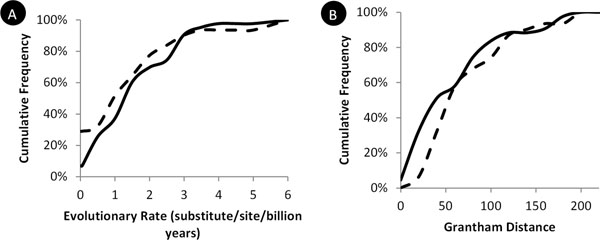
**Comparison between DR-neutral and DR-affecting nsSNVs on evolutionary conservation and biochemical severity.** Cumulative frequency distributions were plotted on evolutionary rate (A) and the Grantham distance (B) for DR-neutral (solid line) and DR-affecting (broken line) variants in DrugVar. Evolutionary rate was estimated using Kumar et al. [27] method applied to the multiple alignments of 46 species obtained from the UCSC Genome Browser [31].

One reason for this ineffectiveness may be that the statistical models for all these tools are trained using Mendelian disease-associated nsSNVs [25,26], which may not be similar to nsSNVs associated with drug responses. This was indeed the case. While Mendelian disease mutations are highly overabundant at evolutionarily conserved sites as compared to common population polymorphisms [23], DR-affecting nsSNVs do not show such strong tendencies (Figure 2a). The average evolutionary rate for positions harboring DR-altering nsSNVs (1.33/site/byr) is not significantly different from that of positions harboring DR-neutral nsSNVs (1.49/site/byr; *P* < 0.6). In addition, the average biochemical severity of nsSNVs implicated in DR-affecting nsSNVs is not significantly different from that for DR-neutral nsSNVs (67 vs. 76; *P* < 0.4, Figure 2b). This is in stark contrast to the pattern observed for Mendelian disease associated variants that show a 50% higher biochemical severity for Mendelian nsSNVs as compared to common population polymorphisms [27]. These observations indicated that the statistical models trained using disease nsSNVs (e.g., EvoD, PolyPhen-2, and SIFT) are not suitable for diagnosing DR-affecting nsSNVs.

### An evolutionary diagnosis model specific to drug-responses

**Figure 3:**
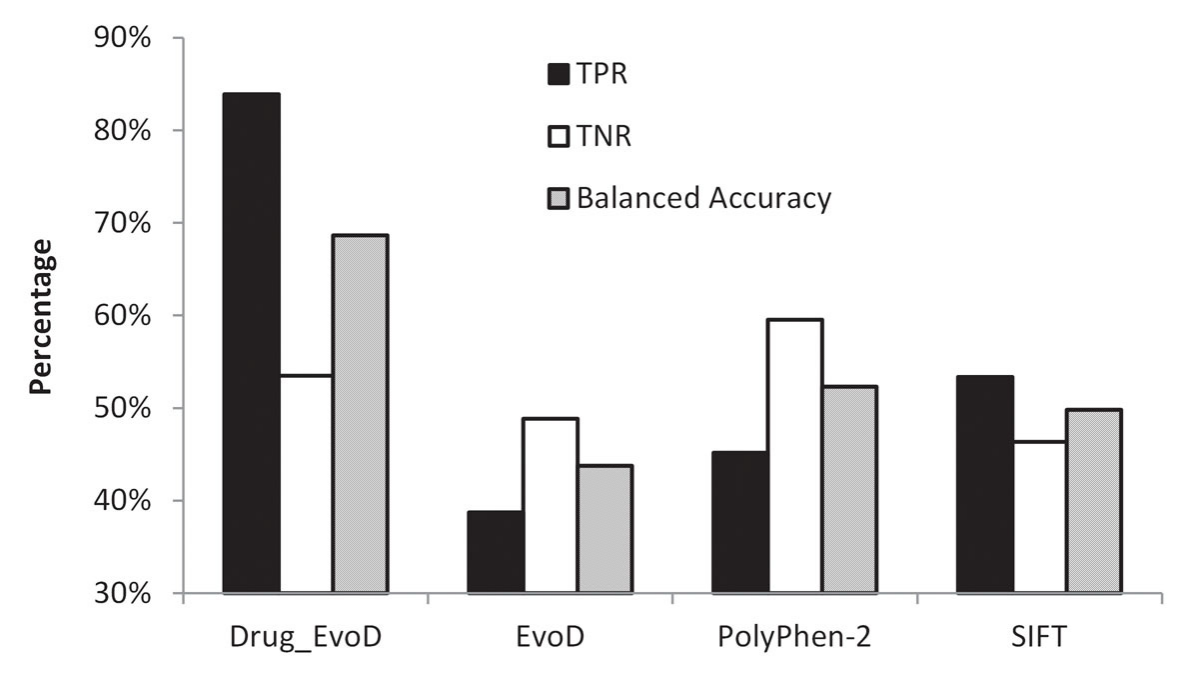
**Performance of Drug-EvoD, EvoD, PolyPhen-2 and SIFT methods tested on the DrugVar dataset.** True positive rate (TPR, solid bars), true negative rate (TNR, open bars), and balanced accuracy (hatched bars) were plotted.

This prompted us to develop a prediction model specifically trained on nsSNVs associated with drug responses (Drug-Evolutionary Diagnosis, Drug-EvoD). We began with 12 evolutionary and biochemical features (see Methods), which were also tested in the original EvoD model [23] that is built for diagnosing disease variants. A feature selection step was applied to identify features that showed a significant power to discriminate between DR-altering and DR-neutral nsSNVs. These features were then used to construct a linear regression model for predictive purposes. This new model (Drug-EvoD) achieved a balanced accuracy of 69%, which is significantly higher than other methods. Such improvement is most likely resulted from an increased true positive rate, which are 84% in Drug-EvoD and 39%-53% in other methods (Table 1, Figure 3). The true negative rate, however, is similar between Drug-EvoD (53%) and other methods (49%-60%).

We then examined the Drug-EvoD model (Table 2) and compared it to the EvoD model [23]. We found that these two models share some fundamental similarities as the coefficients of features are almost always in the same direction. However, significant differences exit. Some EvoD features, such as indel-based entropy, turned out to be not informative in Drug-EvoD. Conversely, some features not in the EvoD model play important roles in Drug-EvoD, such as evolutionary rate estimated among primates and mammals. Even for those features that are shared between these two models, their coefficients are very different. For example, the coefficient of evolutionary rate among vertebrates in Drug-EvoD (-29.5) is only half of that in EvoD at less-conserved sites (-55.2). In summary, we found that the Drug-EvoD model is specifically adapted to differential drug responses and thus achieves better performance than other disease-centered predictive models.

**Table 2:** Coefficients of the 12 features in the Drug-EvoD model.

Parameter	Species	Coefficient
	Primates-only	*12.2*
Evolutionary Rate	Mammals-only	22.3
	All-species	-29.5

	Primates-only	*ns*
Evolutionary Timespan (Position)	Mammals-only	ns
	All-species	*12.1*

	Primates-only	ns
Evolutionary Timespan (Variant)	Mammals-only	*21.8*
	All-species	*-19.9*

	Grantham Value	*-9.8*
Others	Blosum62	*-11.6*
	Entropy2	*ns*

Note –Features that were removed in the feature selection step are indicated by *ns*.

## Conclusions

In this study, we tested the possibility of repurposing computational methods of diagnosing disease variants to predict nsSNVs associated with drug-responses. We found that these tools have rather low accuracies in this domain. This is not surprising because these predictive methods heavily rely on the evolutionary patterns of variants associated with Mendelian diseases, which are under strong purifying selections across species [28,29]. However, we do not expect variants associated with drug responses to share this pattern, as it is unlikely that differential drug responses will lead to significant fitness shift in the long-term evolutionary history of humans. This is supported by our observations that DR-affecting nsSNVs and DR-neutral nsSNVs have similar evolutionary rate and biochemical severity (Figure 2). On the other hand, DR-affecting nsSNVs clearly cause functional changes of proteins, which can be detected using evolutionary data, although it may not show patterns of strong purifying selection.

We then presented a predictive model (Drug-EvoD) specifically trained on nsSNVs with differential drug responses. It was based on a number of features, including long-term evolutionary history of protein positions and alleles, and biochemical severity of variants. Drug-EvoD was the first attempt on exploring ways to identify drug-associated variants with available data. It significantly improves the prediction accuracy upon other models that were developed for identify disease-associated variants. As genetic variants have been found to have a major functional impact on drug response, this kind of computational tools have the potential to give physicians in clinical settings primary information on whether a patient will be subjected to adverse drug response or lack of efficacy, and will help them to prescribe more effective drugs with minimum adverse side effects based on human gene variations.

However, Drug-EvoD was trained on a rather small set of nsSNVs in DrugVar. Although it shows superior performance than existing methods, we believe that such tools will be more reliable with the increase of well-annotated variants and drug response associations. In the future, the accuracy of Drug-EvoD can be further improved with additional features, including 3-D structural models of proteins and drug compounds, which will enable us to predict drug efficiency using molecular docking techniques.

In conclusion, Drug-EvoD represents an initial effort toward computationally modeling the genotype-phenotype relationship on drug responses to make predictions. It demonstrated the feasibility of this new approach. Subsequent methods to improve the size of the control datasets and to construct models with advanced molecular docking algorithms will become invaluable to the research and clinical communities.

## Methods

### Data collection

We used the latest version of PharmGKB [2,15] (accessed on 05/02/2013) to retrieve all available data (filename: var_pheno_ann.tsv) about associations between nsSNVs and their drug response phenotypes, along with information on the drug involved. A total of 1,152 entries were obtained. Redundant entries that corresponded to multiple observations involving the same nsSNVs and the same drug were consolidated into 263 unique nsSNVs in 178 proteins. For each of these nsSNVs, we extracted the type of change and their associated drug efficacy (the ability of a drug to produce a desired effect) and toxicity (abnormal buildup of prescription medication in the bloodstream).

The PharmGKB database presents specific “Strength of Evidence” levels for variant annotations based on clinical results [30]. In the current study, we focused only on variants that have significant clinical annotation level of evidences (Level1A, Level1B and Level2A). That is, variants that were based on a single case report, a study with non-significant results, and studies lacking clear evidence of associations were regarded as having low evidence strength and were not considered. Only variants with high evidence strength in the expert annotations were retained. With this stringent curation and filtering, we obtained 31 distinct nsSNVs in 27 proteins that could be unambiguously used as DR-affecting (true positives, see Table S1 in Additional File 3) and 43 distinct nsSNVs in 36 proteins that could be unambiguously used as DR-neutral (true negatives, see Table S2 in Additional File 4). This composed of the DrugVar dataset. These nsSNVs were cross-referenced on NCBI dbSNP identifers (rsIDs) to retrieve their chromosomal locations in the hg19 human genome build.

### Evolutionary features

For each nsSNV in the DrugVar dataset, multiple species alignments were obtained from the UCSC Genome Browser [31] based on its chromosomal location. Using these alignments, we obtained twelve features for each nsSNV. The first set was the absolute evolutionary rate (*r*) of amino acid change of the position where the nsSNV was found. It quantifies the degree of long-term natural selection against amino acid change and is a measure of the functional importance of the position [27,32-34]. The second set was positional evolutionary timespan (pETS) for each position, which measures the retention of a position over evolutionary time [32-34]. The third set was mutational evolutionary timespan (mETS), which measures the prevalence of an nsSNV at the affected position across multiple species, effectively highlighting the degree of evolutionary neutrality of the mutant. These evolutionary parameters were estimated using 46 diverse species (vertebrates and lamprey), only mammal (36 species), and only primates (10 species) following Kumar et al. [27]. The fourth set consists of biochemical severity of the variant, which was measured using Grantham index [35], and a simple estimate of the amino acid substitution probability as captured in the BLOSUM62 matrix [36]. It also contains indel-based entropy that is the frequency of indels/deletions. Normalization was applied to each feature such that it had a mean value of 0 and standard deviation of 1.

### Drug-EvoD modeling

The statistical model to predict differential drug response for an nsSNV was derived by following the EvoD framework [23]. We used the DrugVar dataset as the training and testing data. The drug responses were regarded as the response variable (D, 100 for positive controls, 0 for negative controls). The 12 features of nsSNVs composed of a feature matrix (F). Within a sparse-learning framework [37-40], we first performed a feature selection step to identify features with significant power to discriminate between DR-affecting (positive controls) and DR-neutral (negative controls) nsSNVs. This step involved minimization of the *l*_1_-norm regularized least square loss using a stability selection procedure that tested a series of regularization parameters [41]. Features that were assigned with non-zero coefficient values in more than 95% of subsamples were retained. For each regularization parameter, we also obtained the weights of corresponding features that best explain the phenotype. If the weight of a feature accounted for less than 5% of the total weight of all features, it was removed due to small effect size. This procedure allowed us to select features that have the most discriminative power between DR-affecting nsSNVs and DR-neutral nsSNVS. In the final model, the weights of selected features were determined that yielded the highest classification accuracy in a balanced subsampling procedure with standard 10-fold cross-validations. This model produced an impact score for each nsSNV. If the impact score is greater than or equal to 50, it was predicted as DR-affecting. If the impact score is less than 50, it was predicted as DR-neutral.

## Competing interests

The authors declare that they have no competing interests.

## Authors’ contributions

SK conceived the study, ZNG and KG performed the analyses, ZNG, LL and SK were all involved in study design, planning analyses, and the interpretation of results. ZNG, LL and SK contributed to writing and revising the manuscript, and all approved the final manuscript for publication.

## Supplementary Material

Additional File 1 **Figure S1.** The distribution of the nsSNVs across different family of proteins. Click here for file

Additional File 2 **“SuppTableforFigureS1.xlsx”** The data in excel format for the distribution of the nsSNVs across different family of proteins. Click here for file

Additional File 3 **Table S1.** The DR-affecting nsSNVs in the DrugVar dataset. Click here for file

Additional File 4 **Table S2.** The DR-neutral nsSNVs in the DrugVar dataset. Click here for file

## Declarations

Publication charges for this article have been funded from research grants from National Institutes of Health (NIH; LM011941-1) and HiCi-1434-117-2 from KAU.

This article has been published as part of *BMC Medical Genomics* Volume 8 Supplement 1, 2015: Selected articles from the 2nd International Genomic Medical Conference (IGMC 2013): Medical Genomics. The full contents of the supplement are available online at http://www.biomedcentral.com/bmcmedgenomics/supplements/8/S1
