# Supplementary material for: Evolutionary Diagnosis of non-synonymous variants involved in differential drug response
Source: BMC Med Genomics. 2015 Jan 15;8(Suppl 1):S6. doi: 10.1186/1755-8794-8-S1-S6 (PMC4315320; doi:10.1186/1755-8794-8-S1-S6)

**Figure S1.** The distribution of the nsSNVs across different family of proteins. (Data can be found in “SuppTableforFigureS1.xlsx”)


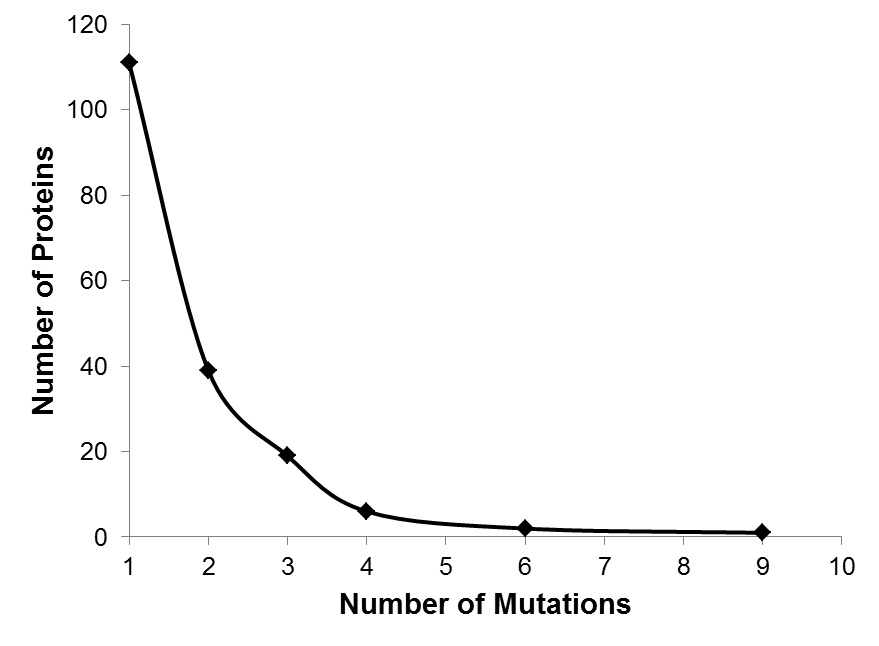

Supplement: Additional File 1 — Figure S1. The distribution of the nsSNVs across different family of proteins. [file 1755-8794-8-S1-S6-S1.docx]
