# Supplementary material for: Evolutionary Diagnosis of non-synonymous variants involved in differential drug response
Source: BMC Med Genomics. 2015 Jan 15;8(Suppl 1):S6. doi: 10.1186/1755-8794-8-S1-S6 (PMC4315320; doi:10.1186/1755-8794-8-S1-S6)
Supplement: Additional File 3 — Table S1. The DR-affecting nsSNVs in the DrugVar dataset. [file 1755-8794-8-S1-S6-S3.docx]

**Table S1.** The DR-affecting nsSNVs in the DrugVar dataset.

| **rsID** | **Gene** | **RefSeq_**  **Protein** | **Variant** | **Drug** |
| --- | --- | --- | --- | --- |
| rs1695 | GSTP1 | NP_000843 | I105V | Platinum compounds, fluorouracil, oxaliplatin, cyclophosphamide, epirubicin |
| rs4680 | COMT | NP_000745 | V158M | Nicotine |
| rs4880 | SOD2 | NP_000627 | V16A | Cyclophosphamide |
| rs4961 | ADD1 | NP_001110 | G460W | Furosemide, spironolactone |
| rs20455 | KIF6 | NP_659464 | W719R | Pravastatin |
| rs25487 | XRCC1 | NP_006288 | Q399R | Carboplatin, cisplatin, oxaliplatin, platinum compounds |
| rs396991 | FCGR3A | NP_000560 | F212V | Rituximab, cetuximab |
| rs1042522 | TP53 | NP_000537 | P72R | Antineoplastic agents, cisplatin, cyclophosphamide, fluorouracil, paclitaxel |
| rs1042713 | ADBR2 | NP_000015 | G16R | Salbutamol, salmeterol |
| rs1050828 | G6PD | NP_000393 | V98M | Chlorproguanil, dapsone |
| rs1056892 | CBR3 | NP_001227 | V244M | anthracyclines and related substances |
| rs1057910 | CYP2C9 | NP_000762 | I359L | Warfarin, antiinflammatory agents, non-steroids, celecoxib, diclofenac, acenocoumarol |
| rs1127354 | ITPA | NP_258412 | P32T | interferon alfa-2b recombinant, ribavirin |
| rs1142345 | TPMT | NP_000358 | Y240C | Azathioprine, mercaptopurine, purine analogues, thioguanine |
| rs1799971 | OPRM1 | NP_000905 | N40D | Ethanol, morphine, naloxone |
| rs1800460 | TPMT | NP_000358 | A154T | Azathioprine, mercaptopurine, purine analogues, thioguanine |
| rs1800497 | ANKK1 | NP_848605 | E713K | Bupropion, antipsychotics, antipsychotics, clozapine, olanzapine, risperidone, ethanol |
| rs1801019 | UMPS | NP_000364 | G123A | Fluorouracil, leucovorin, tegafur |
| rs1801131 | MTHFR | NP_005948 | E429A | Capecitabine, fluorouracil, leucovorin, oxaliplatin, capecitabine |
| rs1801133 | MTHFR | NP_005948 | A222V | Methotrexate, fluorouracil, carboplatin, cyclophosphamide |
| rs1801394 | MTRR | NP_002445 | I22M | methotrexate |
| rs2228001 | XPC | NP_001139241 | Q902K | cisplatin |
| rs2231142 | ABCG2 | NP_004818 | Q141K | rosuvastatin |
| rs2297595 | DPYD | NP_000101 | M166V | Capecitabine, fluorouracil |
| rs3212986 | ERCC1 | NP_036231 | Q504K | Cisplatin, platinum compounds |
| rs3745274 | CYP2A7P1 | NP_000758 | Q172H | efavirenz |
| rs4149056 | SLCO1B1 | NP_006437 | V174A | Simvastatin, cerivastatin, hmg coa reductase inhibitors |
| rs67376798 | DPYD | NP_000101 | D949V | fluorouracil |
| rs75527207 | CFTR | NP_000483 | G551D | ivacaftor |
| rs121434568 | EGFR | NP_005219 | L838R | Erlotinib, gefitinib, carboplatin, gefitinib, paclitaxel, carboplatin, docetaxel, erlotinib, gemcitabine, paclitaxelefitinib |
| rs121434569 | EGFR | NP_005219 | T790M | Erlotinib, gefitinib |
