# Supplementary material for: Evolutionary Diagnosis of non-synonymous variants involved in differential drug response
Source: BMC Med Genomics. 2015 Jan 15;8(Suppl 1):S6. doi: 10.1186/1755-8794-8-S1-S6 (PMC4315320; doi:10.1186/1755-8794-8-S1-S6)
Supplement: Additional File 4 — Table S2. The DR-neutral nsSNVs in the DrugVar dataset. [file 1755-8794-8-S1-S6-S4.docx]

**Table S2.** DR-neutral nsSNVs in the DrugVar dataset.

| **rsID** | **Gene** | **Protein ID** | **Variant** | **Drugs Tested** |
| --- | --- | --- | --- | --- |
| rs1042636 | CASR | NP_000379 | R990G | cinacalcet |
| rs10868138 | SLC28A3 | NP_071410 | Y113C | gemcitabine |
| rs1138272 | GSTP1 | NP_000843 | A114V | Cisplatin, cyclophosphamide, fluorouracil, leucovorin, oxaliplatin, Platinum compounds |
| rs11568658 | ABCC4 | NP_001098985 | G187W | tenofovir |
| rs12233719 | UGT2B7 | NP_001065 | A71S | methadone |
| rs137852808 | CHRNA1 | NP_000070 | C438W | hmg coa reductase inhibitors |
| rs17863762 | UGT1A8 | NP_061949 | C277Y | mycophenolate mofetil |
| rs1799782 | XRCC1 | NP_006288 | R194W | Cisplatin, cyclophosphamide |
| rs1800440 | CYP1B1 | NP_000095 | N453S | Capecitabine, cisplatin, docetaxel, epirubicin, gemcitabine |
| rs1800462 | TPMT | NP_000358 | A80P | purine analogues, mercaptopurine azathioprine, thioguanine |
| rs1800470 | TGFB1 | NP_000651 | P10L | Cyclosporine, mycophenolate mofetil |
| rs1800471 | TGFB1 | NP_000651 | R25P | Cyclosporine, mycophenolate mofetil |
| rs1801158 | DPYD | NP_000101 | S534N | Fluorouracil, leucovorin |
| rs1801282 | PPARG | NP_056953 | P12A | pioglitazone |
| rs2020870 | FMO2 | NP_001451 | D36G | anthracyclines and related substances |
| rs2073618 | TNFRSF11B | NP_002537 | N3K | Pamidronate, zoledronate |
| rs2108622 | CYP4F2 | NP_001073 | V433M | fluindione |
| rs2242047 | SLC28A1 | NP_004204 | R510C | gemcitabine |
| rs2286007 | WNK1 | NP_001171914 | T665I | hydrochlorothiazide |
| rs2290272 | SLC28A1 | NP_004204 | V189I | gemcitabine |
| rs2298771 | SCN1A | NP_008851 | A1056T | Carbamazepine, phenytoin, valproic acid |
| rs2308321 | MGMT | NP_002403 | I174V | temozolomide |
| rs2308327 | MGMT | NP_002403 | K209R | temozolomide |
| rs25489 | XRCC1 | NP_006288 | R280H | Cisplatin, cyclophosphamide |
| rs316019 | SLC22A2 | NP_003049 | S270A | Cisplatin, anthracyclines and related substances,  metformin |
| rs3184504 | SH2B3 | NP_005466 | W262R | Beta Blocking Agents, diltiazem, Thiazides |
| rs34116584 | AGXT | NP_000021 | P11H | Fluorouracil, leucovorin , oxaliplatin |
| rs36210421 | KCNH2 | NP_000229 | R1047L | dofetilide |
| rs3924999 | NRG1 | NP_004486 | R38Q | methamphetamine |
| rs4426527 | AGXT | NP_000021 | I340M | Fluorouracil, leucovorin, oxaliplatin |
| rs45589337 | DPYD | NP_000101 | K259E | Cyclophosphamide, fluorouracil, methotrexate |
| rs487989 | POLA2 | NP_002680 | G583R | gemcitabine |
| rs6312 | HTR2A | NP_001159419 | D49N | Atorvastatin, pravastatin, simvastatin |
| rs660339 | UCP2 | NP_003346 | A55V | antipsychotics |
| rs6759892 | UGT1A10 | NP_001063 | S7A | anthracyclines and related substances |
| rs7080681 | ABCC2 | NP_000383 | R353H | tenofovir |
| rs7483 | GSTM3 | NP_000840 | V224I | Cisplatin, cyclophosphamide |
| rs757110 | ABCC8 | NP_000343 | A1369S | Glibenclamide, glimepiride, sulfonamides, urea derivatives |
| rs763780 | IL17F | NP_443104 | H161R | gemcitabine |
| rs8187758 | SLC28A1 | NP_004204 | Q237K | gemcitabine |
| rs8192678 | PPARGC1A | NP_037393 | G482S | rosiglitazone |
| rs8192924 | CES2 | NP_003860 | R270H | capecitabine |
| rs854560 | PON1 | NP_000437 | L55M | clopidogrel |
